# From Lab on a Chip to Point of Care Devices: The Role of Open Source Microcontrollers

**DOI:** 10.3390/mi9080403

**Published:** 2018-08-14

**Authors:** Trieu Nguyen, Sune Zoëga Andreasen, Anders Wolff, Dang Duong Bang

**Affiliations:** 1 Laboratory of Applied Micro and Nanotechnology (LAMINATE), National Food Institute, Technical University of Denmark (DTU-Food), DK-2800 Kongens Lyngby, Denmark; tring@food.dtu.dk; 2 Department of Micro- and Nanotechnology, Technical University of Denmark, Ørsteds Plads, DK-2800 Kongens Lyngby, Denmark; sunez@nanotech.dtu.dk (S.Z.A.); anders.wolff@nanotech.dtu.dk (A.W.)

**Keywords:** lab on a chip, point of care device, microcontroller, Arduino, open source hardware

## Abstract

Microcontrollers are programmable, integrated circuit chips. In the last two decades, their applications to industrial instruments, vehicles, and household appliances have reached the extent that microcontrollers are now the number-one selling electronic chip of all kinds. Simultaneously, the field of lab-on-a-chip research and technology has seen major technological leaps towards sample handling, sample preparation, and sensing for use in molecular diagnostic devices. Yet, the transformation from a laboratory based lab-on-a-chip technology to actual point-of-care device products has largely been limited to a fraction of the foreseen potential. We believe that increased knowledge of the vast possibilities that becomes available with open source microcontrollers, especially when embedded in easy-to-use development environments, such as the Arduino or Raspberry Pi, could potentially solve and even bridge the gap between lab-on-a-chip technology and real-life point of care applications. The profuse availability and extraordinary capabilities of microcontrollers, namely within computation, communication, and networking, combined with easy-to-use development environments, as well as a very active and fast moving community of makers, who are eager to share their knowledge, could potentially be the difference between a dreadful “chip-in-a-lab”-situation, and the next successful start-up. Here follows a brief insight into how open source microcontrollers could potentially have a transformative effect on the field of lab-on-a-chip research and technology. Details in some specific areas of application are briefly treated before addressing challenges and future perspectives.

## 1. Introduction

**Figure 1:**
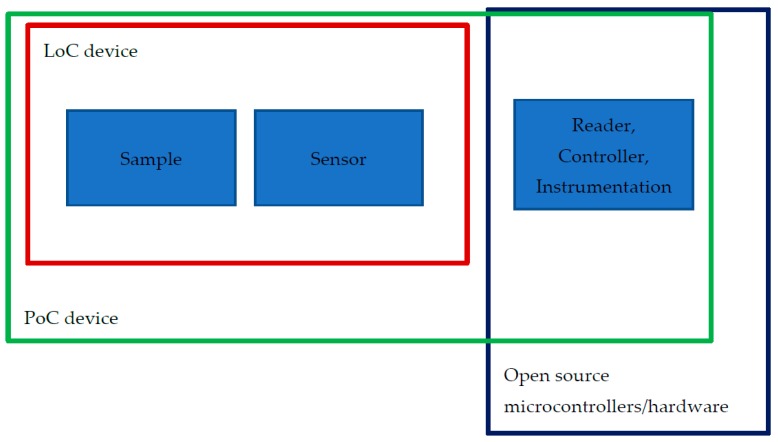
The potential role of open source microcontrollers in the PoC devices.

Lab on a chip (LoC) technology has been a part of the analytical chemistry community since the early 90s [1,2]. The general idea behind this promising area of research, LoC technology, was to achieve chemical analysis of small or ultra-small volumes (in the order of picoliters or smaller [1]) of sample, to reduce both analytical time and cost of reagents, as well as to potentially increase the sensitivity. During the last three decades, not only chemists, but also physicists, biologists, and engineers have put their efforts and resources into fulfilling the original vision of LoC and advanced it to the point where we are now. That is, producing devices, which can operate multiple tasks, from sample preparation to analysis, to fully integrated, sample-to-answer applications, operating on-site, in the form of so-called Point of Care (PoC) applications. PoC devices are here defined as instruments that can perform an analytical or diagnostic test near to the site of interest, for example, a patient in a hospital, at the doctor’s office, or even in the field, in order to provide rapid and on-site results to the operator. The test itself should not only be quick, but preferably also easy to perform, without the necessity of expensive or complicated equipment, and without requiring a specifically trained technician [3,4,5,6,7,8]. Bringing the LoC technology to life through PoC devices is compelling, nevertheless, designing and downscaling to get to the point of portable devices pose many challenges. So far, there have been only a few successes in the transformation of LoC technology to actual PoC devices with real life application [9]. Most of those devices are inherently non-quantitative in their output, only offering either positive or negative answers. Obtaining quantitative answers is generally considered challenging, since it often requires complex and expensive electronics and electrical read-out systems and sensors. This in turn raises the cost, hence opposing the realization of PoC devices [10]. Now, however, we believe that a lot of the potential difficulties and obstacles for obtaining electronic control and read-out have been addressed, and to an increasingly larger extent, solved, in the last few years, due to the rise of the open source community, especially within open source hardware [11,12,13,14,15,16]. In this insight review, we discuss open source microcontroller based platforms, specifically the Atmel-family of microcontrollers used in the incredibly popular Arduino development boards, and the application of the Arduino platform in the field of LoC. The advantage of the Arduino platform is that it comes with three elements, which offer a complete solution for prototyping: the Arduino board (hardware), the Arduino software, and the documentation and learning resources provided by a huge community of makers, who are eagerly sharing their hardware and software solutions on open source platforms, such as GitHub, etc. The Arduino platform allows developers to easily, and with great flexibility, create electronic prototypes, either as stand-alone instruments, or as devices connected to a computer. The Arduino boards read and control a wide range of sensors and output devices, and can be set up to communicate with the software on, for example, a computer, or in a network of devices. Both the Arduino board and its software (the development environment) are easy to use, enabling developers without an engineering background to design and build their own devices and projects quickly and efficiently. We believe that these types of microcontrollers can, to a large extent, fulfill the criteria for creating PoC devices (Figure 1) of existing LoC technologies. Even more importantly, we believe that the (open-source) development environments, as well as the large and easily accessible documentation provided by an ever-increasing community of makers, have reached a state where many, if not most, researchers and technicians advantageously can develop and make their own prototypes of LoC based PoCs, for real-life testing. 


***Brief CV of the authors***


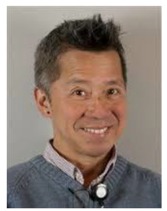



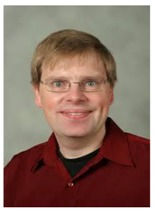



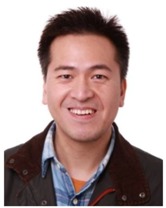



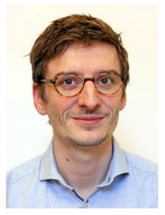

Dang Duong BangAnders WolffTrieu NguyenSune Z. AndreasenProf. Dang Duong Bang received his PhD in 1995 on Molecular biology from Leiden University, Leiden, and the Netherlands. In 2002, he was appointed as senior researcher and leaded the Laboratory of Applied Micro and Nanotechnology (LAMINATE) at National Veterinary Institute, Technical University of Denmark. In 2014, he was promoted as Professor MSO at National Food Institute, Technical University of Denmark. His research focuses on development of total integrated Lab on a chip systems for clinical diagnosis of infectious and food borne diseases. Professor Dang contributed more than 130 papers published in international peer review scientific journals and owned 5 patents.Assoc. Prof. Anders Wolff received his M.Sc. in Chemical Engineering from Technical University of Denmark (DTU) in 1993 and his PhD in Biochemical and Engineering from Delft University of Technology, the Netherlands in Dec 1997. In 1998, Dr. Wolff joined the Department of Micro and Nanotechnology (now DTU Nanotech, DTU, Lyngby, Denmark). In 2000, he was appointed the associated professor and leaded the Cell Handling Group (now BioLabChip) at DTU. His research interests: PCR chip with integrated heaters and thermos sensor, integrated microsystem for sample preparation and DNA amplification. Dr. Wolff owned 7 patents, 80 papers published in international scientific journals.Dr. Trieu Nguyen obtained his PhD (2015) on the microfluidic energy conversion with prof. Jan Eijkel and prof. Albert van den Berg (University of Twente, Netherlands). From November 2015 to January 2017, he worked as a postdoc on microfluidic mixers for protein folding studies in Michigan State University, USA. Currently he is a postdoc researcher at National Food Institute, Technical University of Denmark. His research interests stay on micro-, nanofluidics, Lab on a chip, microfabrication, physical chemistry, electrochemistry and rapid detection of food-borne diseases.Dr. Sune Z. Andreasen received his M.Sc. in physics and nanotechnology from Technical University of Denmark (DTU) in 2013, and a PhD from the same place in 2017 on Lab-on-a-chip devices, specialized on centrifugal microfluidics, electrochemistry, and automated sample preparation. Since then, he has worked as a postdoc at DTU Nanotech developing sensing platforms for biochemical reactions, with emphasis on commercially viable solutions. His research interests include LoC applications, microfluidics, open-source electronics, instrument development and entrepreneurship.

## 2. State-of-the-Art: The Role of Open Source Microcontroller-Based Platforms in Bridging LoC Technology and PoC Devices

As mentioned earlier, microcontrollers are low-cost, single chip computers [17]. The history of microcontrollers can be traced back to the 70’s when they were first developed by Texas Instruments [18]. Arduino (Uno) is a cheap, open source, credit-card-sized, microcontroller board developed by Massimo Banzi and others in 2005 [19], based on a master project made in 2003, by a student under supervision of Banzi. From the very beginning, Arduino was intended to enable artists and designers, who have no or very little knowledge of electronics, to design their own prototypes [20]. Since the introduction as a commercial product in 2005, the Arduino boards and development platform quickly became a phenomenon, spreading internationally, and eventually made popular all over the world. Even though the cost of an Arduino board is only about $20, it has the capacity to interface and control a very broad and diverse range of sensors and actuators (as shown in the 4 examples below and in the instructions in the next section), easily handled via the built-in digital and analog input/output (I/O) pins. These properties (shown in Table 1) make Arduino a perfect and affordable candidate for building electrical read-out applications for LoC systems, and eventually PoC devices. The World Health Organization (WHO) estimates that 70 to 90% of the medical devices donated to the developing countries never worked as intended due to failure during transportation and insufficient training of technical staff [11]. Open hardware platforms, such as Arduino boards, have thereby opened up the opportunity to build medical devices directly in the relevant communities in developing countries for specific case uses, instead of having to be imported from the developed world, hence reducing also transportation cost and training. 

**Figure 2:**
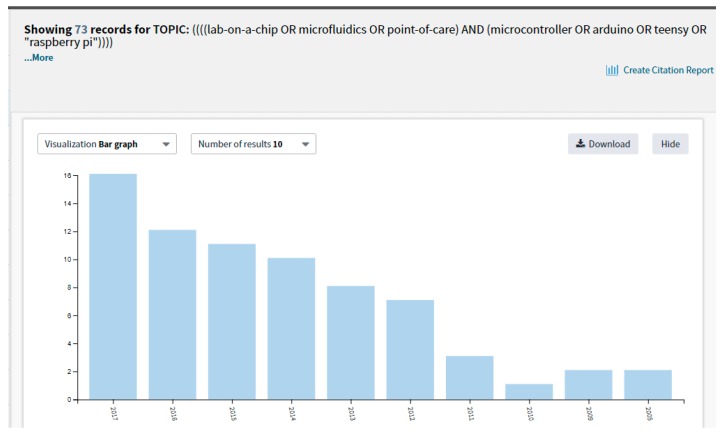
Search result with keyword: “((lab-on-a-chip OR microfluidics OR point-of-care) AND (microcontroller OR arduino OR teensy OR “raspberry pi”))” using Web of Science on 4 August 2018.

We believe that recently scientists in general, and particularly in the field of LoC, have started to notice the raising and beneficial role of open-source microcontroller platforms. For instance, a search using the keyword “microcontroller” in the Lab on a Chip journal gave the results of 31 articles published recently (within the last 2 to 5 years, i.e., from 2013 to 2018). Another search using the keywords “microcontroller” plus “point of care” on Web of Science gave approximately the same result: 32 articles published between 2000 and 2018. A search with the keywords “((lab-on-a-chip OR microfluidics OR point-of-care) AND (microcontroller OR arduino OR teensy OR “raspberry pi”))” in Web of Science gave a result of 73 articles, most of them published recently, from 2010 to 2017, and with an increase in number of articles within the recent year (shown in Figure 2). This result hence seems to confirm our hypothesis that the application of microcontrollers in LoC research labs, and similar places, is about to see a much broader use and application. 

This feature review aims to rise, and hopefully speed up, the awareness in the LoC community, on the beneficial and important role of open source hardware, and especially of microcontroller-based platforms, as we believe we are now at the frontier of the future of PoC devices. Adding to this point, Nature journal reported that there was a conference held in 2016 at CERN, Europe’s particle-physics laboratory near Geneva, Switzerland, with exactly the intention and hope to rapidly increase the awareness about open science hardware solutions to the research community [13].

**Figure 3:**
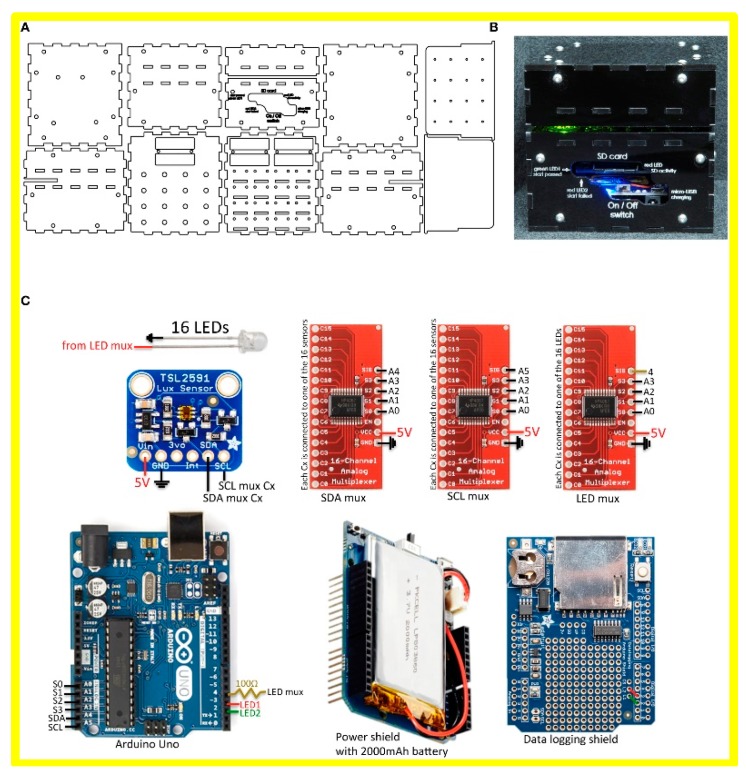
Arduino Uno (marked in the blue square in figure C) used in the work of Pardee, et al. published in Cell 2016 [23]. (**A**) Line drawings used to cut the housing for the electronic reader from black acrylic using a laser cutter. (**B**) Image of the 16-reaction reader from the front. Chip containing paper-based sensors slides into the slot illuminated by the green light. Reader dimensions: 106 mm wide × 116 mm deep × 96 mm high. (**C**) Components and circuit design used to assemble the electronic optical reader. Reproduced with permission from [23].

**Figure 4:**
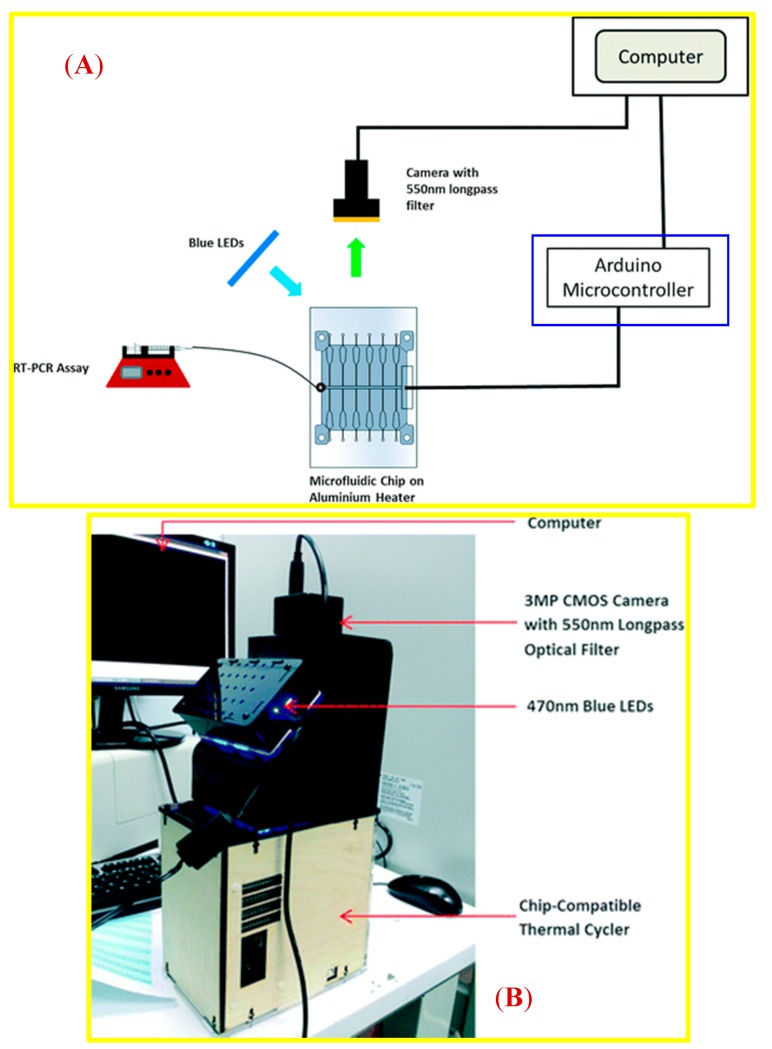
An example of using an Arduino board (denoted in the blue rectangle) for measurement and control temperature in a LoC system (**A**), and as a PoC prototype (**B**). Published in Lab on a Chip journal by Lim et al. Reproduced with permission from [24].

**Figure 5:**
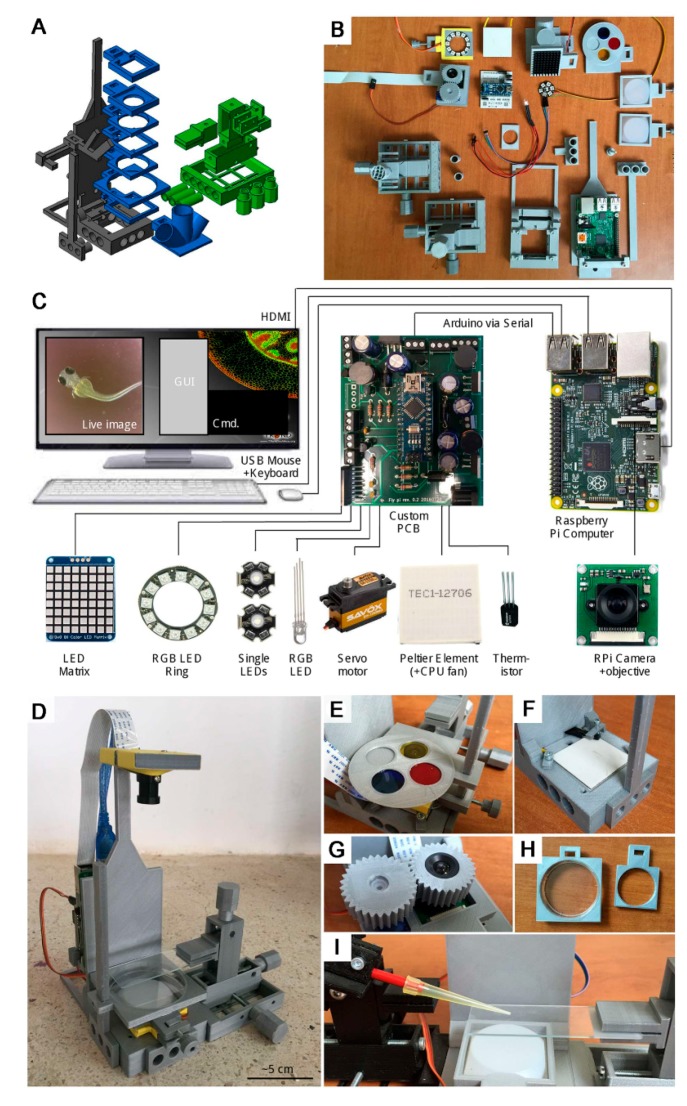
The “FlyPi”/“100€ Lab” platform. Notice the Raspberry Pi and Arduino (on top of custom made PCB) in (**B**) and (**C**). (**A**) The 3D model, colour coded by core structure (black), mounting adapters (blue), and micromanipulator (green). (**B**) Printed parts and electronics, partially assembled. (**C**) Wiring diagram and summary of electronics. (**D**) The assembled FlyPi with single micromanipulator and light-emitting diode (LED)-ring module, diffusor, and Petri dish adapter mounted in the bottom. (**E**) Filter wheel mounted above the inverted camera objective. (**F**) Peltier element and thermistor embedded into the base. (**G**) Automatic focus drive. (**H**) Petri dish mounting adapters. (**I**) A second micromanipulator mounted to the left face of FlyPi holding a probe (here, a 200-μL pipette tip for illustration) above the microscope slide mounted by the micromanipulator on the right. Reproduced with permission from [25].

**Figure 6:**
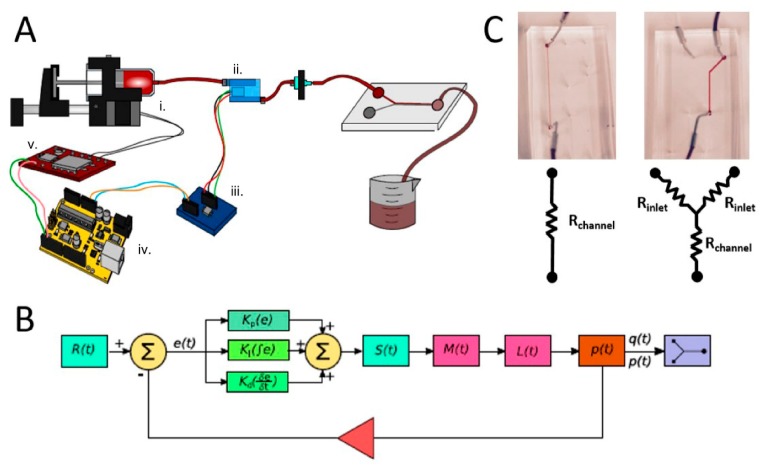
Arduino controlled pressure in a microfluidic system (**A**), through PID or bang-bang algorithm (**B**) to control the desired flow pattern (**C**). Reproduced with permission from [26].

In the field of LoC research, especially when aspiring towards PoC systems, the type of signals that are readable (or otherwise detectable) to the end users is to a large extent based on (but not limited to) capturing of image(s) (especially for cancer screening in the early stage [7,21]) or on sensing fluorescent signal from emitted dyes, which bind on the targets [22]. For illustrating the beneficial role of applying open source microcontrollers on LoC systems to realize PoC devices, we cite four examples of PoC prototypes, employing Arduino development boards to achieve electrical read-out, data recording in a SD card, controlling a LED light source (Figure 3), measuring and controlling temperature (Figure 4), measuring fluorescent intensity (Figure 5), and regulating pressure-controlled microfluidic flow (Figure 6).

Figure 3 shows the application of an Arduino Uno board in a paper-based PoC device used to detect Zika virus, published in the Cell journal in 2016 [23] by Pardee et al. The Arduino Uno was used to controller the LED array (570 nm) and electronic sensors. The total cost of the set-up is under 250 USD. Details on how the set-up is assembled and run are shown in a movie on the publisher’s website.

Figure 4 shows the experimental set-up using an Arduino microcontroller to control a thermal cycler in a real-time polymerase chain reaction (PCR) system, used for microfluidic fluorescence detection. The prototype of the open source PCR can be bought online for 499 USD at http://openpcr.org/.

The third example is the very ambitious “FlyPi”—a “100€ Lab” by A.M. Chagas et al. (2017) [25]. Here, an Arduino Nano connected to a Raspberry Pi computer is located at the heart of a 3D printed open-source platform for fluorescence microscopy and optogenetics, including heat control of the measuring area, see Figure 5. The Arduino Nano is chosen since it is compact and breadboard friendly so that it can be integrated into the custom made PCB as shown in Figure 5. In this application, a combination of a Raspberry Pi and an Arduino board is used. A Raspberry Pi is a very small computer, roughly the size of a credit card. An Arduino board can run one task at a time while a Raspberry Pi can perform multiple tasks. Using microcontroller-based platform to host sensors is an economic way to build up a device, as well as assuring a stable and robust sampling of data, very precise control of actuators, etc. Nevertheless, if more computational power is needed, for example for processing and manipulating of sensor data a microcontroller-based device, like the Arduino, quickly becomes insufficient. That is where a Raspberry Pi comes in. In this example, besides controlling the camera, the Raspberry Pi computer is used to connect with a monitor to visualize the captured images and videos. The obvious inventiveness of the different innovative solutions implemented here, such as fluorescence microscopy using cheap electronic components and theatrical light-coloring films as optical filters, as well as the implementation of Arduino boards for heat and LED control, showcases very well the possibilities of open-hardware solutions for many LoC/PoC applications.

The last example is the Proportional-Integrate-Derivative (PID), (a control algorithm) controlled syringe pump, developed by J.R. Lake et al. [26]. Here, a 3D printed syringe pump is interfaced with an Arduino and in-line pressure sensing hardware (Figure 6). By establishing a feedback loop between the measured pressure and the control of the syringe pump, the pressure in the microfluidic chip can be controlled precisely, and potentially be used in fully automated PoC applications where pressure control is of importance.

## 3. How to Get Started

In order to help beginners to easily get started on building a prototype from scratch, we here outline some guidance. 

The Arduino family of open source microcontroller-based boards is a good starting point for beginners to build their own devices. The Arduino family of development boards include Uno, Nano, Micro, Mega, Mini, Leonardo, etc. See https://www.arduino.cc/en/Main/Products for an overview of all Arduino products and their specifications. Among the Arduino family of boards, the Arduino Uno is the most popular one, and with good reason, as it is both cheap and reliable. The Uno features fourteen digital input/output pins and six analog inputs. Most of the work done in the do-it-yourself community (that requires the use of a microcontroller) has been conducted on the Uno. Using an Arduino Uno hence offers the users a great access to help and support from the community such as the Arduino Forum. If more than fourteen digital I/O are needed, a Mega Arduino board can advantageously be used, as this development board provides the user with fifty-four I/O digital pins. Apart from the development boards, the Arduino platform also features the open-source Arduino IDE (Integrated Development Environment) software, making writing and uploading of code to the boards an easy and simple task. The IDE can be downloaded at the Arduino website https://www.arduino.cc/en/Main/Software#download. In the below section we give some instructions to, as well as provide some references for, controlling of temperature, pressure and movement, using an Arduino Uno.

For temperature controlling and measurement, the Arduino Uno is widely used, for instance in references [27,28,29,30,31,32]. For measuring temperatures lower than 130 Celsius, the ubiquitously available analog temperature sensor TMP36 can be used [32,33]. For measurement of higher temperatures, up to 1000 Celsius, a suitable thermocouple and break-out board containing temperature sensing electronics can be selected [31,34]. For building of a thermocycler such as a polymerase chain reaction (PCR) machine, it is informative to look at the work of Chai, a company founded by Josh Perfetto and Jessie Ho at http://openpcr.org where they built up a PCR thermocycler based on an Arduino Uno. The source code and full list of components are available at their website, and it is possible to buy a completely open source PCR for 499 USD at their website. For the PID library used to control temperature with an Arduino, the most popular one seems to be the one developed by Brett Beauregard, and the software library is available for download from http://brettbeauregard.com/blog/2011/04/improving-the-beginners-pid-introduction/ or https://github.com/br3ttb/Arduino-PID-Library/blob/master/PID_v1.h.

For creating and controlling of precise movements, an Arduino Uno can also be used to control one or more stepper motors. A stepper motor is a special type of motor that moves in discrete steps. A stepper motor is therefore a good choice for a project requiring very precise movements, such as a 3-D printer (ILIOS HD kit for instance [35]), or a micro-movement stage as in references [36,37] where respectively two and three stepper motors are used. For controlling stepper motors, AccelStepper library will come handy, see for example reference [38].

Furthermore, stepper motors controlled by an Arduino Uno can also be used to generate and control pressure in a microfluidic system, as shown in example 4 in the previous section. IDE code and bill of materials can be downloaded from the website provided in reference [26]. Alternatively, an Arduino Uno can also be used to create a desired waveform for a certain duration of time to control a peristaltic pump as shown in reference [39].

Table 2 summarizes some major websites and sources of information, such as online lessons, tutorials, e-books, as well as places for buying electronic components. Forums for discussion and getting help from the Do-It-Yourself (DIY) community are also listed. 

## 4. Challenges and Future Perspectives

The first challenge is that the beginners who try to build a device based upon an open source microcontroller may feel the task overwhelming. Nevertheless, a little effort invested to learn the basics of how to handle an open source microcontroller, such as an Arduino board, will quickly pay off many times over, as beginners start to build up their own devices and projects. Another challenge is that equipment and devices based on other people’s open source hardware solutions, often are still at an early stage of a product’s evolutionary process. Commercially bought devices hence may provide longer product life times and more robust calibrations/functionality. Nonetheless, as more ideas and designs are contributed, the degree of complexity of open-source devices will grow fast. Eventually, not only the scientific community but also the public will benefit due to an increased availability of low-cost, yet highly sophisticated, open source devices.

Some PoC devices have been fully commercialized (QuickVet, https://www.smb.dk/smb/quickvet), some are still in the academic research stage (SMARTDIAGNOS, http://www.smartdiagnos.eu/). In each stage, the challenges are obviously different, yet we have no doubt that the opportunities of achieving success on those PoC devices are now within reach for much larger parts of the LoC research field, due to the rise of open source communities, especially the ones based on open source hardware, such as the Atmel microcontrollers of the Arduino boards. As Isaac Newton once wrote: “If I have seen further it is by standing on the shoulder of Giants.” (in a letter to his rival, Robert Hooke, written in 1676) [40]. In the scientific world nowadays, open source hardware (and software) already is considered “a giant’s shoulder”, as suggested by Dryden in an article published in Analytical Chemistry last year (2017) [16]. The Arduino boards and software is one of the most successful and well-recognized open hardware platforms [11]. Together with all reasons aforementioned in the previous sections, we hence believe that microcontroller-based development boards (Arduino, Raspberry Pi, etc.) will aid in closing the gap between in-lab LoC technologies and real-life PoC devices. Furthermore, as smartphones are now becoming increasingly inexpensive, easy to use, and globally available, microcontroller-based hardware can easily be made to connect to smartphones via for example Bluetooth or Wi-Fi. Ultimately, this could lead to the possibility of online monitoring and online detection applications of the PoC devices, for instance by combining cheap, microprocessor-based, equipment, with the ubiquitously available smartphones. Furthermore, together with the rapid development of 3D printing and other additive manufacturing techniques, combined with increasingly cheap prototyping of electronics, for example, light emitting diodes (LEDs), phototransistors, potentiostats, and so on, will speed up the prototyping process and create more opportunities for low-cost and sensitive instrumentation and for realizing PoC devices [14].

## Figures and Tables

**Table 1:** Common properties of open source microcontroller platforms and PoC devices.

	Point of Care Devices	Open-Source Microcontrollers (Arduino Platform)
Low cost	√	√
Easily accessible	√	√
Easy to use	√	√

**Table 2:** List of resources to help the beginners to get started.

Type of Resource	Company’s Name	Webpage	Remarks
**Books and Video lessons**	Safari Books Online	safaribooksonline.com	One can create a trial account in order to download books and video lessons on Arduino, Raspberry Pi in 10 days. This is a great source of information.
**Tutorial**	Udemy instructors Programming electronics academy	https://www.udemy.com https://programmingelectronics.com/arduino-tutorials-all/	Plenty of Tutorials on Do-It-Yourself (DIY) using Arduino, Raspberry Pi, Python…
**Forum and code library**		https://github.com/ https://forum.arduino.cc/ www.instructables.com	
**Where to buy**			
	RS Components	www.rs-online.com	
	Sparkfun Electronics	https://www.sparkfun.com/	
	Adafruit Industries	https://www.adafruit.com/	
	Farnell element14	https://farnell.com	
	Mouser Electronics	www.mouser.com	
	DigiKey Electronics	www.digikey.com	
	Conrad Electronics	https://www.conrad.com	
